# No More Evasion: Redefining Conflict Behaviour in Human–Horse Interactions

**DOI:** 10.3390/ani15030399

**Published:** 2025-01-31

**Authors:** Emily O’Connell, Sue Dyson, Andrew McLean, Paul McGreevy

**Affiliations:** 1 Independent Researcher, 959 Bacchus Marsh Road, Bullengarook, VIC 3437, Australia; 2 Independent Researcher, The Cottage, Church Road, Market Weston, Diss IP22 2NX, UK; sue.dyson@aol.com; 3 Independent Researcher, 3 Wonderland Ave, Tuerong, VIC 3915, Australia; andrewmclean@esi-education.com; 4 Sydney School of Veterinary Science, Faculty of Science, University of Sydney, Sydney, NSW 2006, Australia; paul.mcgreevy@sydney.edu.au

**Keywords:** equitation science, resistance, disobedience, ridden horse behaviour, learning theory, ethology, pain, anthropomorphism, anthropocentrism

## Abstract

Euphemisms, anthropomorphisms, and equivocation are established characteristics of traditional equestrian language. ‘Evasion’, ‘resistance’, and ‘disobedience’ are common labels assigned to unwelcome equine behaviours, implying that the horse is at fault for not complying with the human’s cues and expectations. These terms appear to overlook multiple motivations that may directly result in the horse offering unwelcome responses, which may then inadvertently be reinforced. This article revisits some of the anthropocentric inferences in these terms and explores the harmful consequences of such convenient but incorrect labels before proposing a redefinition of ‘conflict behaviour’ in human–horse interactions: Responses reflective of competing motivations for the horse that may exist on a continuum from subtle to overt, with frequencies that range from a singular momentary behavioural response to repetitive displays when motivational conflict is prolonged. Addressing how inadequate terms may mask pain, obscure the horse’s motivation, and deflect human culpability, this commentary highlights the merits of a multidisciplinary approach to terminology across equine research. Acknowledging that variables contributing to behaviour can be biological, environmental and anthropogenic, it emphasises the need for more investigation into the relationships between equicentric motivations reflecting equine telos and problematic horse behaviours.

## Simple Summary

Unwanted horse behaviours in human–horse interactions are often labelled with historical terms such as evasion, resistance, and disobedience, which fail to recognise current knowledge of the horse’s nature, as well as its mental and sensory abilities. These common labels often inadvertently place the horse at fault without acknowledging the many mechanisms that may be affecting or motivating the behaviour shown. If we are to continue riding horses, it is imperative that we endeavour to protect them from mental distress and pain. This commentary discusses the multiple influences on equine behaviour in human–horse interactions and proposes a multidisciplinary redefinition of the term ‘conflict behaviour’ for human–horse interactions that encompasses this complexity. Our proposed definition is as follows: Responses reflective of competing motivations for the horse that may exist on a continuum from subtle to overt, with frequencies that range from a singular momentary behavioural response to repetitive displays when motivational conflict is prolonged. We suggest that by using this redefined term and recognising the primary contributing factors involved in undesirable equine responses, future research can continue to determine how best to interpret the possible causes of unwanted behaviour.

## 1. Introduction

Equestrianism is under increasing scrutiny as its social license to operate is challenged by both the lay public and stakeholders within the equestrian and racing industries [1,2]. With still images and video footage of ridden horses being shared rapidly and globally, it is becoming increasingly important that there is a consensus about what constitutes appropriate behaviour, what is abnormal, maladaptive or simply undesirable, and what the potential explanations for problematic behaviour may be [3]. Within the equestrian industry, there has been a tendency to normalise coercive corrections of problematic behaviour and to ignore or overlook the motivations behind these behaviours. The terminology used is often vague and misleading and fails to consider the reasons why the behaviours occur and the motivations underpinning them. Historically, there has been a failure to recognise the roles of learning [4], confusion and pain [5] in the emergence and persistence of unwelcome behaviour. This may reflect the legacy of limited knowledge of equine learning theory among the horse-owning public at all levels, including riders, professionals [6,7], coaches [8], and clinicians [9,10,11].

The aim of this commentary is to discuss the common anthropocentric and anthropomorphic terms that characterise historical and current terminology in the light of current knowledge of learning theory (learning processes), biomechanics, and current understanding of horses’ possible reactions to pain. We consider the range of biological, environmental and anthropogenic variables that influence equine behaviour in interaction with humans, such as when being handled, ridden, or driven. Then, we question whether the umbrella term ‘conflict behaviour’ can be updated to provide a cohesive approach across disciplines of equine research. In essence, by highlighting the strengths and limitations of methods for observing and determining causes of problematic equine behaviour, we emphasise the need for a multidisciplinary approach to investigating the links among biological, external, and anthropogenic equine motivation, not only for the horse’s welfare but for the sustainability of human–horse interactions.

## 2. Problematic Behaviours in Human–Horse Interactions

While alterations in the behaviours of horses can give us some insight into their current arousal and affective state, it does not necessarily follow that we can easily distinguish the motivations behind these behaviours by merely observing them. Nonetheless, repetitive displays of abnormal behaviours would not be considered typical for horses that are free from pain and distress, such that they have their basic physiological and psychological needs met and have been appropriately trained according to the principles of learning theory [5]. We note that a possible exception to this is the presence of emancipated stereotypies. Within human–horse interactions (for example, handling, riding, driving), however, problematic equine behaviours are often labelled with terms that are anthropocentric, and these often fail to recognise the intertwined variables that may motivate and influence behaviour.

## 3. Anthropomorphism and Anthropocentrism

Anthropomorphism describes the attribution of human characteristics and intentionality to non-humans. It has been hypothesised that humans default to this practice due to the human brain’s evolution to facilitate the processing of social information, such as behaviour or facial features [12]. Hence, it is likely that the attribution of mental state and motivation to all animals, including horses, is an inherent trait that acts to explain the cognitive processing of others, even when it may not take place. Anthropocentrism, however, describes the belief that human beings are the central or most important entity on the earth, interpreting the world entirely through human values and sensory experience [13]. Through this commentary, anthropomorphism and anthropocentrism will be referred to, and although they are not interchangeable in meaning, the reader is encouraged to remember that both often reflect a failure to recognise the animals’ telos (the hardwired nature of the animal) and umwelt (the animals’ perception of the world around them based on species-specific sensory abilities).

When anthropomorphism is involved in qualitative behavioural assessments that may indicate affective states [14], it may play a part in revealing both positive and negative welfare. However, most assessments involve subjective interpretations of the data, as humans can only imagine what it is like emotionally for the animal involved. It is imperative here to acknowledge that anthropomorphism itself is not ipso facto problematic, and arguably, there is evidence to suggest that along with empathy and attachment, it is one of the basic components of the human–animal relationship [15]. However, to assume the motive driving a particular behaviour without consideration for an animal’s biological nature and needs alongside its mental and sensory abilities is ill-informed and arguably egocentric. To reject anthropomorphic descriptions of or reasonings for behaviour is not to imply that horses cannot experience feelings and emotions, nor is it to de-individualise or objectify them. It is widely accepted that mammals such as horses are sentient [16] even though their level of self-awareness may be beyond empirical measurement.

Anthropocentrism is especially likely to compromise the treatment of horses when humans misinterpret equicentric motivations behind problematic behaviours and respond to them in anthropocentric ways [17] that overlook their telos. It often obscures equine motivation and can inadvertently result in compromised welfare, where the horse is blamed for failing to comply with anthropogenic cues. For example, when a horse does not respond to a conditioned cue to accelerate, it may be deemed to be resistant or disobedient. More accurately, flaws in reinforcement contingencies that result in pressure cues escalating to the point of pain [4] or the presence of pre-existing underlying pain may account for these behaviours [18].

Urquiza-Haas and Kotrschal [12] argue that reliance on anthropomorphism progressively decreases when one adopts alternative reasoning to explain problematic behaviours. Subsequently, the horse’s protection from the maladaptive consequences of anthropomorphism and anthropocentrism relies on increased knowledge of their telos, umwelt, cognitive abilities, behaviour, motivations and the continued education of all humans working with them. Although anthropocentric explanations of horse behaviour have prevailed for millennia, equitation science is interpreting the mechanisms and processes of welcome and unwelcome equine responses through an ethological evidence-based lens, revealing the mechanisms of optimal practice in horse training [19]. Equitation science privileges the most parsimonious ethological explanation of equine responses at liberty, in-hand and under-saddle.

## 4. Evasion, Resistance, and Disobedience

The anthropocentric words evasion, resistance and disobedience are found recurrently in horse riding, training, and handling manuals [20,21,22,23,24,25], the popular equestrian literature, as well as equine health and welfare journal articles [26,27,28,29]. However, most authors do not offer definitions of these terms in the publication, an omission that renders the terms open to misinterpretation. Ethologically, these terms are inaccurate when describing a horse’s behavioural motivation because they are predicated on the erroneous assumption of cunningness and premeditation, which are higher-order cognitive processes unavailable to horses and thought to be unique to humans [30]. It has been argued that ‘the problem with these terms is that they imply malevolent and calculated behaviour on the part of the horse’ and that such a flawed approach fails to acknowledge that horses lack the ability to reason [4]. In addition, from a scientific perspective, assuming higher mental processes without proof represents an unacceptable rejection of the null hypothesis.

Of course, there are exceptional accounts of horses learning complex tasks with seemingly approximate goals that initially elude explanation. For example, there are videos of horses learning to open the latch of stable doors to release conspecifics [31]. Such tasks are motivated by the horse’s social nature and can emerge from chained responses, but once fully formed, prima facie, they appear to show insight and inadvertently fertilise further beliefs of a horse’s ability to be calculating and malevolent, which, in turn, can cause unrealistic expectancies during training. To assume that a non-human animal knows the difference between right and wrong according to human morals and opinions is to misinterpret the behaviours in question and the adaptive motivation behind them. If horses cannot calculate and premeditate these behaviours as inferred by terms such as evasion, resistance and disobedience, we must aim to identify what motivations and mechanisms underpin these behaviours and use terms that accurately reflect learning processes and the possible array of potential motivations. Doing so allows one to unpack the three-term contingency (ABC) of antecedent, behaviour, and consequence [32]. It also facilitates an understanding of how a given response may be reinforcing because it lessens pain. We would like to acknowledge here that in some languages, evasion and avoidance translate into the same word. Due to this, some may not distinguish evasion as problematic as a descriptive term as disobedience or resistance.

Within the Fédération Equestre Internationale (FEI)’s *Dressage Handbook: Guidelines for Judging*, evasion, resistance, and disobedience are all defined (see Table 1). However, these definitions are not currently accessible on the FEI website and exist only within a hardcopy book [33], which increases the likelihood of misinterpretation and multiple definitions. It is stated within this hardcopy that these guidelines do not replace the rules but, instead, are aimed at improving communication related to judging, riding, and training [33,34]. However, resistance and disobedience feature throughout the 2024 FEI Dressage Judging Manual [35], the FEI Object and General Principles of Para Dressage [36], 2024 FEI Dressage Rules [37], FEI Para Dressage Rules [38], 2024 FEI Driving and the 2024 FEI Para Driving Rules [39]. Within these, the definitions and examples of such resistant or disobedient behaviours, if given, are vague and left open to interpretation. When compared to the Oxford English dictionary definitions of these terms, one can easily appreciate how these words nourish the beliefs of a villainous or naughty horse within equestrianism.

When analysing the FEI’s definition and use of any of these terms in documentation, the anthropocentricity becomes apparent. There is an absence of any attempt to nominate possible motivating causes of problematic behaviours such as ‘refusing to go forward’ [39]. Such failure to respond to accelerator cues may arise from physical difficulty doing what is asked due to possible injury, pain, or discomfort. Similarly, there may be a physical/conformational limit or overdevelopment of some elements of the musculoskeletal system and underdevelopment of others. Alternatively, confusion and distress may contribute to such behaviours, including when, for example, there have been historic simultaneous clashes of opposing aids such as acceleration and deceleration, errors in a rider’s timing of the application and release of aids/cues and a horse’s discrimination of these.

Importantly, the anthropocentric examples of evasions presented in the FEI Dressage Handbook [34] include ‘tilting the head, open mouth, broken neckline’. These are unwanted behaviours likely to be influenced by physical constraints such as bit pressure and restrictive nosebands, as well as perhaps being obscured, especially to the eye of the observer, if they are predominantly focused on other performance indicators [40]. Although a regulation of noseband tightness with a FEI standardised tool is anticipated for all disciplines in 2025 [41,42], the FEI Dressage Rules still vaguely state that the tightness of a curb chain on a double bridle must not result in harm to the horse [37]; yet, nowhere within these rules is it described what the FEI constitutes as harm nor how that would be measured; instead, this is left up to interpretation. Possible unintended and largely unseen harms could include tongue pain, bruising, ischaemia [43], oral lesions [44], and learned helplessness [4]. The risk of these outcomes may increase because of the mechanical advantage that arises from the lever effect of the curb bit, resulting in a lighter feel of rein tension by the riders than the pressure the horses encounter in their mouths.

Even though the FEI’s rules describe evasions, resistances, and disobedience as undesirable, recent studies [26,45] provide evidence that some of the behavioural signs described in the rules are going unnoticed by dressage judges. These behaviours arise despite the popularity of physical constraints, e.g., restrictive nosebands (for which the rules are yet to be updated to align with current evidence). Such constraints may effectively mask a horse’s motivational conflict, discomfort, and pain to the extent that manifestations of a horse’s discomfort are repeatedly missed by dressage judges [46]. Paradoxically, in other animal performance sports such as rodeo, scores, instead, depend on these conflict behaviours being present and the human’s ability to cope with them [47].

If the equestrian sports industry continues to use language that infers that a horse knows better than to display problematic behaviour, the consequent welfare of the horse will be poor. It fails to acknowledge the motivational conflict that the horse must be experiencing to display such behaviour, as well as the influence this negative affective experience has on the horse’s mental and physical health. If equestrian sports are to maintain their social license to operate, it is vital that the use of language reflects an understanding that includes and encompasses the telos and umwelt of horses. As language consequently influences human behaviour towards animals [48], transparency is essential to meet ideal industry standards. Deconstructing language is a crucial step towards shifting the relationship between equestrianism and members of the public towards sustainable horse sport but, perhaps more importantly, in improving the human–horse dynamic [49] and horse welfare. As recently highlighted by Hall and Kay [50], a good life for domesticated horses relies on human behaviour; our ability to meet their physical and emotional needs, provide mostly positive affective experiences for them and correctly interpret their behaviour.

**Table 1:** Historical and current definitions and use of ‘evasion’, ‘resistance’, and ‘disobedience’.

**Text**	**Definition of evasion**	**If no clear definition is given,** **examples within the text**
2007 FEI Dressage Handbook, Guidelines for Judging [34]	Avoidance of the difficulty, correctness, or purpose of the movement, or the influence of the rider, often without active resistance or disobedience (e.g., tilting the head, open mouth, broken neckline, etc.). Bit evasions are means of avoiding correct contact with the bit.	-
2024 Oxford English Dictionary [51]	To escape or avoid (someone or something), especially by guile or trickery.	-
2024 FEI Judging Manual [35] & 2024 FEI Dressage Rules [37]	Anticipation or precipitation of the movement, resistance to or evasion of the contact, deviation of the hindquarters from the straight line, spreading or inactive hind legs and dragging forefeet are serious faults.	-
	Resistance to or evasion of the Athlete’s hand, being either “above the bit” or “behind the bit”, demonstrate lack of submission.	
2024 FEI Para Dressage Rules [38] & 2024 FEI Object and General Principles of Para Dressage [36]	-	Anticipation or precipitation of the movement, resistance to or evasion of the contact, deviation of the hindquarters from the straight line, spreading or inactive hind legs and dragging forefeet are serious faults.
		Resistance to or evasion of the Athlete’s hand, being either “above the bit” or “behind the bit” demonstrate lack of submission.
2024 FEI Driving and Para Driving Rules [39]	-	(13. Rein back) The Horse must remain on the bit, be straight in the body, not evade or resist the contact, and the poll should remain the highest point.
2024 FEI Eventing Rules [52]	-	-
Equitation Science 2nd Edition [53]	Descriptive terms for conflict behaviours where evasions are similar to resistances, except that evasions refer to the more severe and violent behaviours. These terms arose because of the horse’s natural tendency to avoid pressure/pain by learning through negative reinforcement to perform any attempted behaviour that results in lessening of pressure/pain. The problem with these terms is that they imply malevolent and calculated behaviour on the part of the horse, whereas these behaviours are more likely to be the result of errors in negative reinforcement.	-
	**Definition of resistance**	**If no clear definition is given,** **examples within the text**
2007 FEI Dressage Handbook, Guidelines for Judging [34]	Physical opposition by the horse against the rider. Not synonymous with disobedience nor with evasion. Can be momentary or pervasive.	-
2024 Oxford English Dictionary [51]	The dislike of or opposition to a plan, an idea, etc.; refusal to obey.	-
2024 FEI Judging Manual [35] & 2024 FEI Dressage Rules [37]	-	By virtue of a lively impulsion and the suppleness of the joints, free from the paralysing effects of resistance, the Horse obeys willingly and without hesitation and responds to the various aids calmly and with precision, displaying a natural and harmonious balance both physically and mentally. All of these criteria enable the Athlete to follow the movements of the Horse smoothly and freely or react appropriately if the Horse shows tension or resistance of any kind.
2024 FEI Para Dressage Rules [38] & 2024 FEI Object and General Principles of Para Dressage [36]	-	Putting out the tongue, keeping it above the bit or drawing it up altogether, as well as grinding the teeth or agitation of the tail, are mostly signs of nervousness, tension or resistance on the part of the horse. Any resistance which prevents the continuation of the test longer than twenty (20) seconds is punished by Elimination. However, resistance that may endanger Athlete, Horse, Officials or the public will result in elimination for safety reasons earlier than within twenty (20) seconds. This also applies to any resistance before entering the Dressage arena. Resistance may last no longer than sixty (60) seconds. However, resistance that may endanger Athlete, Horse, Officials or members of the public may result in elimination for safety reasons earlier than within sixty (60) seconds.
2024 FEI Driving and Para Driving Rules [39]	-	A horse is considered to offer resistance if, at any time and for whatever reason it refuses to go forward (with or without moving back), turns around, rears.
2024 FEI Eventing Rules [52]	-	-
Equitation Science 2nd Edition [53]	Descriptive terms for conflict behaviours where evasions are similar to resistances, except that evasions refer to the more severe and violent behaviours. These terms arose because of the horse’s natural tendency to avoid pressure/pain by learning through negative reinforcement to perform any attempted behaviour that results in lessening of pressure/pain. The problem with these terms is that they imply malevolent and calculated behaviour on the part of the horse, whereas these behaviours are more likely to be the result of errors in negative reinforcement.	-
	**Definition of disobedience**	**If no clear definition is given,** **examples within the text**
2007 FEI Dressage Handbook, Guidelines for Judging [34]	Willful determination to avoid doing what is asked, or determination to do what is not asked.	-
2024 Oxford English Dictionary [51]	Refusal to obey.	-
2024 FEI Judging Manual [35] & 2024 FEI Dressage Rules [37]	-	“Severe resistance, disobedience throughout” as reason for marks of 1, 2 or 3.
2024 FEI Para Dressage Rules [38] & 2024 FEI Object and General Principles of Para Dressage [36]	It is considered to be a Disobedience when:	Representing After a Disobedience: After being penalised for a refusal, run-out or circle, an Athlete, in order to make another attempt, is permitted to circle one or more times without penalty, until the Athlete again presents their Horse at the obstacle.
	(a) Athlete attempts to pass through an Obstacle and their Horse shies away from the Obstacle at the last moment without hitting any part of the Obstacle. (b) The Horses run away, or, in the opinion of the President of the Ground Jury, the Athlete has lost effective control. (c) The whole turnout comes to a complete halt, with or without stepping back anywhere on the course, in front of or in an Obstacle, or a Multiple Obstacle, with or without knocking down any element. (d) Not passing through an Obstacle with the whole turnout, running out of a Multiple, circling within a Multiple or reining back by the Athlete between start- and finish line.	
2024 FEI Driving and Para Driving Rules [39]	-	Any resistance in the forward movement, kicking or rearing is considered to be disobedience.
2024 FEI Eventing Rules [52]	-	-
Equitation Science 2nd Edition [53]	-	-

## 5. Conflict Behaviours

Equitation science [53,54] generally refers to problematic behaviours of ridden horses as ‘conflict behaviours’. From a purely ethological perspective, conflict behaviours are those which arise due to being in a state of conflicting motivation, and neither the term nor the manifestation of conflict behaviours is unique to horses [55]. It is important to emphasise that the word ‘conflict’ in conflict behaviour does not describe any agonistic relationship between the animal and its environment, but instead, the battle within the animal itself due to competing or conflicting motivations [56]. Like all animals, equids have evolved mental processes to deal with these opposing contingencies, and when an avoidance motivation is involved in the conflict, the behaviours that arise occur extremely quickly and are often resistant to elimination [57,58]. This potential reaction speed is an adaptive mechanism to respond first rather than pausing to evaluate the suspected threats, a useful evolutionary response often referred to as the ‘Life-Dinner Principle’ [59].

Table 2 depicts a selection of definitions that exist for conflict behaviour, from equitation science, veterinary science, and ethology, highlighting the similarities and differences between them. It includes the in-depth definition from the *Encyclopedia of Applied Animal Behaviour Science and Welfare*, and whilst this is a remarkable source of information, it is not practical for easy application nor easy use in equestrianism. Despite being a positive step away from the anthropocentric laced terms of evasion, resistance, and disobedience, many of the equitation science definitions of conflict behaviour understandably focus on operant contingencies and the use of aversives. That said, the majority of equitation scientists emphasise that pain should be ruled out before conflict behaviours are remediated with training approaches alone [60]. This is important because pain may render trained responses unpleasant and lead to a learned aversion to personnel by association that may manifest as aggression [61]. In contrast, veterinary definitions appear to emphasise the possible role of biological variables, such as pain, in eliciting identical behaviours. This is reasonable for clinicians, concentrating on the importance of eliminating pain as a contributor to the conflicting motivations that may underpin a behavioural outcome. However, we would like to briefly draw attention to the notion of coping within the Federation of Veterinarians of Europe (FVE) definition in Table 2, as coping behaviour is ‘performed with the aim of reducing the effects of an aversive stimulus on the fitness of an individual’ [62]. Whilst we can appreciate the necessary importance of addressing aversives, it must not be overlooked that the conflicting motivations of conflict behaviour can also involve appetitive/pleasant stimuli. It is imperative to delve further into all the possible and likely interacting causes of conflict behaviours rather than to dismiss them as being the same as coping mechanisms, be they successful, unsuccessful, adaptive, or maladaptive.

We feel that there is merit in updating and streamlining the term conflict behaviour to reflect the original concept of conflict in ethology and provide communication ease between disciplines and into equestrianism.

## 6. Contributing Variables in Problematic Ridden and Driven Behaviour

**Figure 1:**
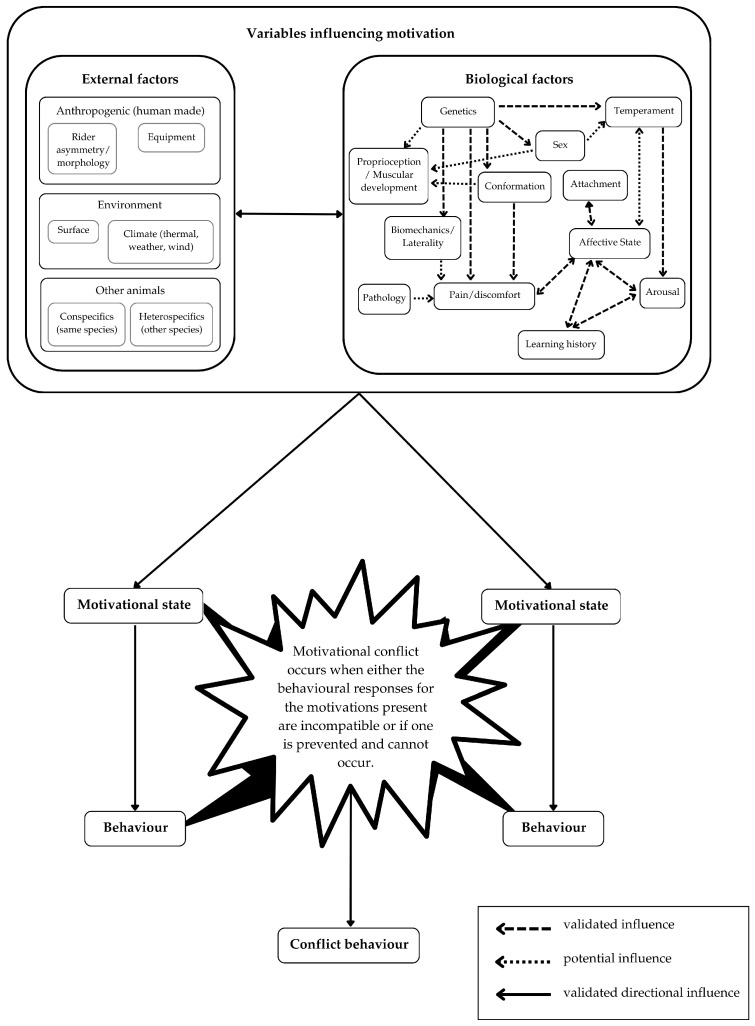
Equine behavioural responses to conflicting motivations—a conceptual model. The arrows present, whilst speculative within the context of this innovative framework, were generated based on validated evidence of influence.

Behavioural responses in mammalian species are commonly used as indicators of arousal and affective state. However, responses can range from a single reflex response (such as to noxious stimuli in nociception) to complex behaviours that require higher processing, such as learning [67]. At present, the definitions of biological variables such as pain, stress, and emotional state and the measurement of such cannot be directly relied on with animals because we cannot quantify exactly how animals feel, nor can they communicate to us verbally how they subjectively experience the world [67]. In addition to this, when responses reflect a level of evaluation and choice by an animal, they are demonstrating behavioural complexity that, in turn, makes it challenging to infer specific causation (see Figure 1).

Motivations that prevail during human–horse interactions need to be explored further, especially because they may compete and be subject to flux during a single episode of training. This will help to provide a roadmap rooted in robust science for all involved (owners, riders, coaches, veterinarians, manual therapists, tack fitters, rule makers, rule enforcers, judges, and spectators) to identify and evaluate what may be going on for the horse. We must be careful not to automatically deduce cause from behaviour, especially within ridden or driven behaviour, because to do so ignores the likelihood that out-of-context behavioural responses to mental stress may be evident. Furthermore, it causes a disservice to the seminal work in which equine science and equitation science are founded (for example—ethology, psychology, veterinary medicine, anatomy, biochemistry). Improving the ability of owners, riders, and practitioners to identify the motivations triggering problematic behaviour and the mechanisms of learning theory that explain undesirable outcomes occurring would have a positive effect on horse welfare and human safety and in protecting all equestrian sports’ social license to operate. Being mindful of the need to avoid assuming a singular cause from behaviour, we see conflict behaviour as a helpful term that reminds observers that motivations can be complex, conflicting, and covert.

The causation of problematic behaviours such as pain may be elusive, and the difficulty in noticing them has often been dismissively attributed purely to the horse being a prey animal, needing to mask pain or discomfort from potential predators. However, as Carbone [68] argues, this logic would suggest that pain is easily identifiable in all predatory species; yet, studies of behavioural responses in a multitude of species show that many predatory species also suppress obvious signs of discomfort in the presence of humans. Whilst the presence of humans may well influence a horse’s behaviour, including when in pain, there are many reasons for an animal to modify their behaviour, and it is far too one-dimensional to attribute this entirely to the domestic horse having evolved from a wild prey animal. Although this evolution occurred, with many biological adaptations of behaviour, such as avoidance being innate [69], this reasoning seems to offer an unsatisfying explanation for the continued failure by humans to recognise the multiple influences on behaviour in the domestic horse. Given the genetic alterations to the horse through the selection pressures of domestication for over four millennia [70], horses integrating with humans are probably subject to many more motivating factors, even if problematic behaviours emulate those also displayed by wild equids. This is not to suggest that factors such as pain and fear are not significant motivators of behaviour, but instead, to acknowledge that there may well be multiple motivators present for the horse and that within this incompatibility, problematic behavioural responses occur.

## 7. Biological Variables

Biological variables influencing equine behaviour include all those internal to the horse. Some of these, such as temperament [70] and conformation, have been scientifically acknowledged for decades as having an influence on the outcomes of human–horse interactions, while knowledge of others is only in its infancy. Here, we have chosen to discuss the variables of pain and psychological state as these have often been overlooked in discussions of the ontogeny of behaviours [5,71].

### 7.1. Pain

Pain is a universal experience in the animal kingdom and is understood to be an evolved adaptive response that helps to protect the organism’s body from potential or actual tissue injury [72]. In essence, pain is a protective stress response. Lundblad [73] assessed facial expressions in horses and concluded that stressful situations can result in facial expressions in horses akin to facial expressions of pain. In addition, the phenomenon of pain is dynamic and subject to individual variation, as well as having temporal and contextual effects. So, the assessment of pain can be subjective, and while most human assessments can self-report pain, this is not possible in animals, so we cannot directly measure an animal’s experience of pain [74]. Instead, we must infer pain from the animal’s behaviour and, in some instances, from physiological changes such as heart rate, heart rate variability, respiratory rate, and hormonal markers such as changes in cortisol concentration. It is, however, acknowledged that changes to these are not always exclusively in response to pain [75]. In humans, both acute and chronic pain have been reported in the absence of harmful stimuli (for example, allodynia), and due to this, pain is now defined as an ‘unpleasant experience that has both sensory and emotional components’ [74]. It would be unwise to assume that aspects of this evolved process are absent in non-human animals, and consequently, the emotional and physical facets of pain are now often acknowledged as distress [76]. Although currently difficult to measure empirically, these conceptual components are critical in understanding how pain may manifest in horses.

Given that behavioural changes can indicate the possible presence of pain in human neonates [77] and adults with dementia [78], one would predict that the same applies to animals [79]. Pain-based behavioural markers may be especially observable when the animal is encountering acute pain due to harmful stimuli causing nociception because this reaction bypasses the perception filters of the animal, which may inhibit or magnify some sensations of pain [80], taking the message directly to the spinal cord [72]. Importantly, horses share a similar distribution of cutaneous nociceptors as humans [81], which suggests that neurologic pain reception is similar in the skin of horses and humans.

The current commentary is focused on behavioural responses, so it is important to emphasise the limitations involved in using behavioural indicators to evaluate emotional and physical distress. A study of dogs and cats showed that critical changes in behaviour may be so subtle that they are noticeable by only the carer or another person very familiar with that individual animal [75]. In comparison, an equine study showed that many horse owners were unaware of their horses showing abnormal behaviour whilst being tacked up [82], highlighting the essential need for improved education in the equestrian sector. It is imperative to determine when a horse is in pain, even though the manifestation of pain may be influenced by learning, emotional components, and equipment.

Horses’ behavioural responses to pain and fear occasionally appear similar [83], showing evasive or flight responses to reduce exposure to the painful stimulus. We note that these responses may also be in response to additional stressors being present and eliciting fear [50]. This is similar to findings that several facial expressions used to identify pain in horses were also present in fear-provoking conditions [84]. Pain during interactions with humans may result in anticipation of pain (and resultant fear) during future interactions with that individual or in similar circumstances [85]. Bucking and rearing are potentially dangerous behaviours to humans. Bucking can be an expression of joie de vivre, post-inhibitory rebound, motivational conflict, or pain [60], but when bucking is repetitive, pain is the most common cause [86]. Therefore, it is understandable that veterinarians should exclude pain before the horse begins a programme of behaviour therapy [53].

In addition, there may be a causal dilemma here when it is difficult to determine what came first: the pain that caused the behaviour or a behaviour that caused pain. There is a growing body of evidence that numerous changes in ridden horse behaviour may indicate the presence of musculoskeletal pain or other sources of pain, such as oral lesions [3,18,27,28,44], but we are also only in the infancy of understanding the psychological factors influencing equine behaviour and the causal relationships among such factors [85]. The relationships between pain and emotion have overlapped through evolution in order to generate complex biological mechanisms that protect the individual from perceived and real danger, and this highlights the importance of the psychological state of the animal when attempting to interpret an observed behaviour [72]. The introduction of objective gait analysis has raised questions about the clinical significance of measurable asymmetry in movement patterns [87]. Concurrent assessment of ridden horses’ behaviour may enable differentiation between pain-related asymmetry versus inherent laterality.

### 7.2. Psychological State (Arousal, Affective State, Temperament, and Attachment)

#### 7.2.1. Arousal

With its evolutionary origins as a prey animal, the horse has a sensitive arousal response to avoid predation. So, even in a domestic context, an aversive event, whether pathological, environmental, or anthropogenic, has the capacity to trigger this ancient adaptive response, often resulting in escape behaviours [53]. Like all animals, the horse has evolved higher mental processes to deal with any conflicting contingencies that arise, and often, when an avoidance motivation is involved in the conflict, the ability to escape an aversive prevails over other motivations [55]. The amygdala plays an important role in the mammalian brain, and it is involved in motivation, emotional memory, and learning [88]. As horses have a comparatively enlarged amygdala compared to that of other domesticated species [88], it could be argued that they are relatively sensitive to negative reinforcement learning. In other words, they may have a particularly acute need to avoid physical and mental discomfort.

Stress is a process that demands emotional or physiological responses for the individual to regain a state of allostasis. Distress occurs when this is ongoing [89]. A horse with chronic musculoskeletal pain is more likely to show problematic behaviours under saddle that have historically been labelled evasions, resistances, or disobedience [5]. This is similar for a horse in mental distress, as the magnetic resonance imaging of mammalian brains shows that both pain and stress responses in the hippocampus are remarkably similar [89]. Further investigation into the escalation from stress to distress caused by the accumulation of aversives [90] will be valuable in revealing the components involved in problematic behaviour and ultimately improving safety in interactions for both horses and humans.

#### 7.2.2. Affective State and Temperament

Despite there being little published research on moods and affective states on horses, McBride and Mills [85] have discussed the impacts of these psychological factors on equine performance and training, stating that ‘both mood and emotional [/affective] states are crucial in determining how the horse perceives and reacts to its environment’. So, it could then be hypothesised that mild or chronic distress may be directly influenced by the individual animal’s temperament (genetically based stable predisposition to reactivity), mood (temporary state of average affective states), and affective state (short-lived stimulus response emotional reaction) [85]. If this is the case, animals with higher scores of neuroticism may be more sensitive to and less tolerant of aversive events, and those scoring lower appearing ‘stoic’ in comparison. It has been preliminarily observed in dogs that extroverted individuals express their pain more noticeably [91]. Additionally, horses with higher extroversion show more resistant (as labelled within the study) behaviours and attempts at avoidance and escape [92]. The relationship between behaviour, physiological biomarkers, and psychological states such as affect, mood, and temperament requires further analysis [93]. This, alongside the impact on behaviours expressed in conjunction with pain, could also improve safety in human–horse interactions.

#### 7.2.3. Attachment

Horses are social animals, and their motivation for group cohesion can be overwhelming. For example, separation distress is commonly seen in horses. In addition, as a reinforcing stimulus, group cohesion is highly prioritised by horses and is usually more motivating than food [94]. Interestingly, in a study of attachment theory in domestic horses [95] it was revealed that while horses show proximity seeking, they did not always choose their carer as a primary attachment figure. Perhaps this can be explained by the social nature of horses and the prospect that the group is more important than the individual. Similarly, another (pilot) study on the effect of fear-eliciting stimuli on the human–horse relationship revealed that the horses also showed a similar disregard for individual human familiarity following aversive exposure [96]. On the other hand, there is ample evidence that horses’ heart rates can be lowered when certain skin zones targeted for intraspecific allogrooming are stroked by humans [97,98].

Human–animal relationships are complex and are modulated by the sum of competing extant motivations, including satiation, as well as arousal and affect. Therefore, it has been suggested that although the provision of food or other resources can facilitate the development of a positive human–animal relationship, it cannot ipso facto explain all of what is regarded as horse–human attachment. [99]. Similarly, the presence or absence of behaviours described as evasion, resistance, and disobedience is likely to be influenced by some aspects of attachment and the overall human–animal relationship. However, there is likely to be a more prosaic explanation for the appearance of such behaviours.

The relevance of the social ethogram to the human–horse dyad and its limitations in explaining horse behaviour when horses are threatened or cornered has been reviewed elsewhere [60]. Horses can be expected to have a negligible attachment bond with humans who are interacting with them in ways that do not align with their social programming. This is especially important for veterinarians and farriers.

#### 7.2.4. Learning History/Experience

An animal’s learning history/experience encompasses their experience with others, their environment, and events throughout life. Learning represents processes in which experience adjusts an individual’s behaviour [63]. An animal’s learning history and life experience represent a dynamic set of variables, which can have strong negative influences on the affective state and learning itself when involving confusion, fear, or pain [50,100,101]. In addition, within a domestic context, management such as group housing [102] and forage provision [103] has been suggested to positively influence the human–horse interaction. This highlights the significance of the hours experienced by the horse outside those interacting with humans [50]. The influence on the horse’s learning history/experience within a human–horse interaction is discussed further in the use of learning theory by riders and handlers (see ‘External variables’).

## 8. External Variables

External variables influencing horse behaviour include any current aspects of the environment in which the horse and human interact, such as footing [104] and climate conditions [105]. It also includes all of the human-influenced aspects, such as the rider/handler themselves (for example, rider asymmetry, morphology), their use of learning theory in training, and all equipment used on the horse. External variables may be aversive simply because of the direct negative experience they generate, their novelty, or because of associations with previous negative outcomes.

### The Importance of Use of Learning Theory by Rider/Handler

The underpinnings of some interactions between horses and humans can be traced back, very broadly, to the ethological origins of the dynamics between predator/prey or conspecifics [106]. The horse has evolved with the sensory and mental processes, musculoskeletal system, and behaviour to respond to certain aversive events. In equitation, negative reinforcement learning is the mechanism most commonly used to train ridden responses [19]. That is, an aversive pressure (examples include tactile interactions, noise or human presence) is applied and then removed as soon as the desired response is displayed. It is the removal of the aversive stimulus in negative reinforcement that is critical to bringing a response under stimulus control, as is clear in the theory of reinforcement [107]. Despite the problems associated with poor application of negative reinforcement, in optimal practice, it maintains the horse’s limbs under stimulus control and, to a large extent, even while competing against the motivation to flee in the face of a perceived threat [108].

The use of negative reinforcement renders horses vulnerable to errors in training and the application of aversive pressure. One reason for this is the fine line between the optimal arousal (stress) level required to learn and the higher levels that inhibit learning and cause distress [108]. Unfortunately, many current practices and techniques in training ridden horses apply aversive events to the extent that they may damage tissues, inhibit learning, and induce pain or stress, all of these outcomes compromising welfare [4]. Fear responses can be installed quickly due to ‘one-trial-learning’, a process in which an innate response that removes an aversive stimulus at the first trial can become a strengthened learned response after only a single presentation. One-trial learning is especially likely if the aversive event involves neuropathic pain or fear [57]. Importantly, it is also worth highlighting that to be ethical in animal training, the aversive stimulus should be no more than an irritant that the individual wants to avoid instead of causing fear or pain [108]. Unfortunately, the quadrants of operant conditioning (negative reinforcement, positive reinforcement, negative punishment, and positive punishment) are often misunderstood by horse owners [4] and the equine-focused scientific community [109]. It is clear that these four quadrants of operant conditioning operate alongside the context of the so-called three A’s (arousal, affective state, and attachment) [110]. Affective state is hypothesised to underpin an animal’s decision-making, providing moment-to-moment prediction [111]. So, as Perone [112] suggests, there is merit in focusing on the affective outcomes of training procedures rather than the four quadrants of operant conditioning when comparing contingencies of behaviour.

## 9. Complexity of Contributing Variables

**Figure 2:**
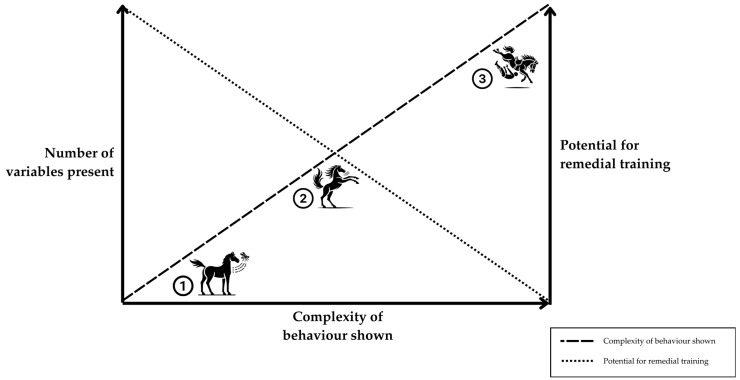
The relationships among the variables influencing equine behaviours, complexity of behaviour shown and the potential for remedial training of unwanted behaviour. Example 1: Horse swats light touch off body with tail—a simple, instantaneous reflex response to noxious stimuli (nociception). Example 2: Horse rears in paddock in response to novel stimuli, and the influence of post-inhibitory rebound before moving away from novel stimuli—an innate avoidance behaviour. Example 3: Horse bucks under saddle, unseating rider—typically an unwelcome behaviour in the human–horse interaction. Note: This diagram and examples are offered as figurative scenarios and have not yet been validated with empirical studies. The arrows present, whilst speculative within the context of this innovative framework, were generated based on validated evidence of influence.

Conflict behaviours during direct contact with humans inherently involve more variables and may be especially difficult to remediate, even once pain is eliminated (see Figure 2). It is possible to see an inverse correlation between the complexity of stimuli and the potential for remediation. In an anthropogenic environment, horses may habituate to a minor tactile irritant (such as from fly-spray) through repeated exposure or through purposeful desensitisation techniques. As stimuli become more complex, the horse at liberty may become habituated or sensitised through exposure to the stimuli, resulting in decreased or increased reactivity. However, when one adds the increased complexity of a human–horse interaction and conflict behaviour, the potential for re-training declines. Compared to the examples without a human–horse interaction, this instance presents complex relationships among multiple factors, and, as a direct consequence, remedial training may be complicated due to the challenge of dissecting these.

## 10. Conceptual Frameworks and Technology

There is a growing number of tools and models that capture different facets of animal behaviour, management, and training, and this commentary does not propose to provide an exhaustive review of them. Table 3 displays the attributes of various published observational instruments for identifying and/or assessing behaviour in domestic equids, including their potential for use in human–horse interactions and their utility for logging conflict behaviours.

Established conceptual frameworks may offer ways to approach Tinbergen’s four questions to explore: function; evolution; causation; and development [120], but in addition, more recent models explore how core affect (the two-dimensional emotional framework of valence and arousal [121]) influences the individual animal’s decision-making [111]. Furthermore, tools such as those developed by the online resource Canine Arthritis Management (CAM) [122] provide veterinarian advice and guidance to owners when observing, assessing, and acting on their dog’s behaviour changes. Feasibly, similar tools could be developed for horse owners because evaluating patterns of [55,123] and the diversity of animal behaviour [124] are understood to be beneficial in measuring mental and physical well-being.

These days, technologies are developing fast, and it is hoped that some will directly improve the comfort of horses when they are ridden [125]. Several gait assessment tools using sensors and cameras have been developed for use in a clinical setting [126,127], and other wearable technologies, such as heart rate monitors [128], rider symmetry monitors [129], rein tension meters [130], and behavioural monitoring tools [131], are being used in equine research, as well as becoming commercially viable as training and surveillance tools for owners and healthcare providers. Additionally, with the advent of machine learning and artificial intelligence (AI), despite the capacity for monitoring and evaluating equine behaviour being currently in its infancy [132], it may be that technology will be preferred to methods that rely upon human observation. AI models such as that described by Feighelstein et al. [133] that include deep learning software, which takes input video footage to automatically recognise horse emotional states from facial expressions, hold great promise.

Additionally, there is a growing appreciation of the Bayesian brain hypothesis, which acknowledges the brain’s function in curating expectations based on past experience to develop predictions of a human’s decisions. This construct has proven critical in current understandings of human cognition and affect. It may also reveal insights into how animals’ affective states influence their responses and the welfare consequences of these responses [111,134]. These theories promise to address how an animal’s perception can be shifted by its experience and how experiences involving fear or pain might render the individual vulnerable to cognitive deficits, such as compromised capacity to re-learn [111,134]. Although in its infancy within animal welfare science, this perspective promotes the importance of a positive affective state during training, as is highlighted in the 2020 Five Domains model of animal welfare assessment [135]. It aligns with the published use of the model to reveal the negative consequences of some equitation practices [136].

Technological advances could have a pivotal role in sustaining horse sports [137]. For example, it has been shown that dressage judges, limited by human cognitive capacity, visually prioritise focal areas rather than processing the performance in toto [40]. In the future, horse sports such as dressage may well be analysed in real-time by AI software to support judges’ observations [138]. Such technological advances could promote the detection of positive equine behavioural expressions, generate further objectivity, and provide transparency within equestrian sport.

## 11. Methods Used in Measuring Behaviour

Until technology effectively usurps the human eye to ensure that we overcome inherent biases, we will continue to rely on direct observations or behavioural scoring of video. Experienced equestrians are accomplished at assessing their horses to make management and healthcare decisions from group composition to daily ration formulations. Similarly, humans have been attributing reason to horse behaviour under the saddle since ancient Greece [139]. However, equitation science provides an evidence-based interpretation of various desirable and undesirable outcomes in-hand and under-saddle. For the behaviours of non-verbal subjects to be measured, they must be directly observed [140]. Ethograms are an inventory of behaviours shown by a species and are invaluable when observing behaviour. They must describe each response in a way that allows for objective observation and identification [120]. As it is often difficult to interpret and determine the cause of an observed behaviour, ethograms are fundamental in the study of domestic horse behaviour, whether through the equitation science or veterinary science lens. As proposed by Pierard, et al. [141], there remains a need for a standardised descriptive ethogram of ridden horse behaviour, and it could be argued that it should include all behaviours displayed in human–horse interactions, for example, those of handling, lungeing, long-reining, and driving. Many published scientific studies contain ethograms of specific suites of behaviours, but they rarely use a shared nomenclature and often include a combination of structural, functional, or causal descriptions [141]. It is imperative that scholars agree on the range, function, and structure of the behaviours shown by an organism before they apply Tinbergen’s four questions to explore the biological significance of conflict behaviours.

## 12. Conflict Behaviour Revisited

**Figure 3:**
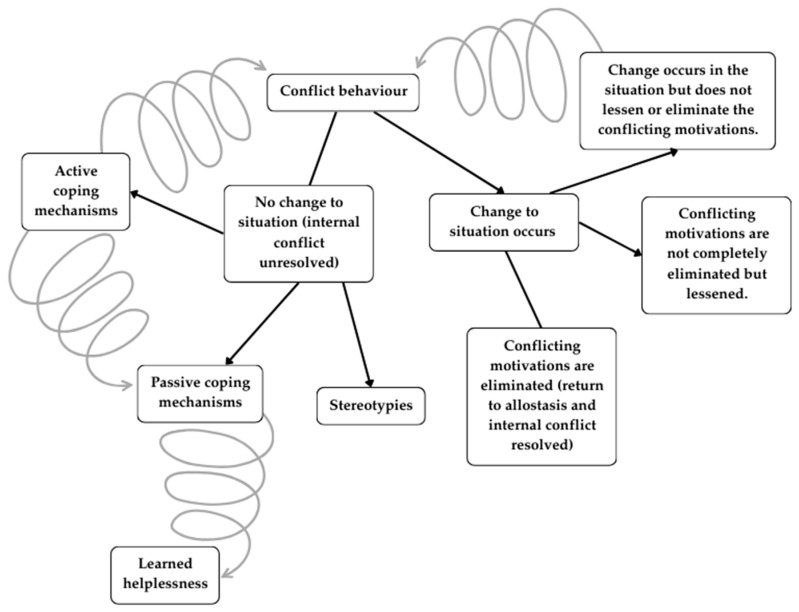
The outcomes of conflict behaviour and the consequences for the horse—a conceptual framework/roadmap. This diagram is offered as a figurative scenario and has not yet been validated with empirical studies. The spiral arrows are a conceptual approach to the spiral/stacking nature of affective experiences on behaviour presentation. Whilst speculative within the context of this innovative framework, the aspects were generated based on validated evidence of influence.

When the multiple demanding motivations a horse faces are incompatible or if one is prevented and cannot occur, the horse enters motivational conflict, and this is when ‘conflict behaviour’ occurs (see Figure 1). Conflict behaviour, therefore, exists on a spectrum and will inevitably intersect with other lexicons of equine behaviour; however, these terms can exist simultaneously. Despite not being interchangeable, many of these behavioural terms are not mutually exclusive when used. Figure 3 illustrates these associations and the escalation/de-escalation that can occur when the behavioural response does not result in an altered condition for the individual.

Active/proactive coping mechanisms (characterised by flight or fight behaviours) may result in the behaviours spiralling as the animal becomes highly sensitised to the conflicting motivations. In contrast, passive/reactive coping mechanisms occur when the internal conflict is persistent and cannot be resolved, such as when active coping mechanisms are thwarted. Through the process of habituation, responses become less frequent and alongside a negative affective state, this can spiral into learned helplessness. Stereotypies generally occur when there is a deprivation of a biologically hardwired need (such as freedom of movement, foraging or socialisation). Displacement and redirected behaviours may occur when a change to the situation occurs and lessens the motivational conflict but does not completely remove it. Furthermore, conflict behaviours become subject to operant conditioning when a change in the situation arises in response to the display of them. This is how conflict behaviours may become established over time and repeated even when the motivations present are removed (see ‘Change to situation occurs’ in Figure 3).

There are many ways that behaviours may be labelled, and we do not suggest a dichotomous solution. Instead, we want to highlight the complexities of the motivations behind the behaviour and its expression, especially within a human context. By further streamlining the definition of conflict behaviour, we are optimistic that it may travel into the equestrian discourse without significant misinterpretation despite the intricacies of behaviour being so complex.

Ultimately, we propose that the term ‘conflict behaviour’ in the context of a human–horse interaction is redefined in veterinary science and equitation science as follows:

Responses reflective of competing motivations for the horse that may exist on a continuum from subtle to overt, with frequencies that range from a singular momentary behavioural response to repetitive displays when motivational conflict is prolonged.

## 13. Conclusions

Reflecting on the way we talk and write about horses both in academia and sport reveals the potential for a clearer roadmap towards better protecting horses in our care. Equestrian training is under scrutiny, now more than ever, with the recent discourse of questionable methods used in the training of sports horses. In the face of growing concern for equestrianism’s social license to operate and advances in technology that may characterise horses that are fit for purpose in various pursuits, the need for clarity in reporting problematic behaviour is pressing. The intended purpose of this commentary in offering a revised definition alongside the accompanying discourse is to encourage equitation science, veterinary science, and equestrians to collectively embrace the use of conflict behaviour in labelling unwanted behaviour in human–horse interactions. To do so is to prioritise the horse by appreciating the complexities that anthropogenic variables add to the presentation of behaviour. While some may see this endeavour as semantic, a transparent and cohesive approach is needed to interpret common ‘horsey’ euphemisms scientifically and to better educate horse owners and riders. To keep riding horses, we have a responsibility to safeguard them against pain, mental distress, and unethical training practices. The redefinition of conflict behaviour offered here encompasses the frequent complexity of conflicting motivations for horses when interacting with humans. Future research should continue to investigate how best to decipher the possible causes of conflict behaviour in the light of the fresh definition whilst still acknowledging the other contributing variables that may be involved. With this, we hope the equitation science and equestrian discourse can now refer to conflict behaviour and reject the terms evasion, resistance and disobedience.

## Figures and Tables

**Table 2:** A selection of seven definitions of conflict behaviour that arise in the applied animal behaviour and welfare, ethology, equitation science, and the veterinary behavioural medicine literature. Examples have been excluded from these definitions to facilitate comparisons. Similarities across these definitions are highlighted using different formatting, with **conflicting/incompatible motivations in bold,** *aversive stimuli in italics* and appetitive/pleasant stimuli underlined.

**Source**	Definition (in Full * or Abridged †)
The Encyclopaedia of Applied Animal Behaviour and Welfare [62]	† Conflict behaviour describes one category of stress-induced **behaviour changes that arise from conflicting motivations**, especially when escape/avoidance responses are not consummated. It can appear as a set of responses of varying duration that are usually characterised by hyper-reactivity…. When arising from a relatively brief *exposure to a single stressor*, these responses typically present as hyper-reactive behaviours. On the other hand, when conflict behaviours arise from chronic exposure to conflicting stimuli, responses can manifest as repetition and ritualization of original conflict behaviours…. conflict behaviours may have evolved as adaptive processes to resolve stressful situations. The term conflict behaviour is appropriate to both interspecific and intraspecific interactions.
Elements of Ethology [63]	* In ethology, the term ‘conflict’ is used to mean **conflict between behavioural tendencies** rather than conflict between animals. Such **conflict between motivational systems** will arise frequently in animals.
Equitation Science 1st Edition [54]	† A set of unwelcome responses of varying duration that are usually characterized by hyper-reactivity and arise largely through confusion… and are thought to have their origins in *intraspecific agonistic behaviours* and *counter-predator responses.*
Equitation Science 2nd Edition [53]	† Stress-induced **behavioural changes that arise from conflicting motivations**, especially when avoidance reactions are prevented. If the stressor is recurrent, conflict behaviour may become repetitive and ritualised. In equitation, conflict behaviours may be caused by application of simultaneous opposing signals (such as go and stop/slow), such that the horse is unable to offer any learned responses sufficiently and *is forced to endure discomfort from relentless rein tension and leg pressures.* Similarly, conflict behaviour may result from incorrect negative reinforcement, such as reinforcement of inconsistent responses or lack of pressure removal.
International Society for Equitation Science (ISES) definition from the proceedings of the 1st symposium [64]	† Conflict behaviours may be caused by *opposing signals being applied by the rider if the horse attempts to escape enduring pressures*, from one cue being indistinguishable from another or from the incorrect use of negative reinforcement, such as the reinforcement of incorrect responses, no removal of pressure for the correct response, or no shaping of responses.
Federation of Veterinarians of Europe (FVE) joint position paper on the animal welfare implications of animal behavioural modification, training methods, and ability to express species-specific behaviours [65]	† Stress-induced **behaviour arising from conflicting motivations**, behaviours arising from an inability to cope with *mental or physical discomfort…*
Merck Sharp and Dohme Veterinary Manual [66]	† Conflict arises when a pet has **competing motivations** or is motivated to perform more than one opposing behavior. This might occur when a dog is motivated to greet but is fearful of approach, perhaps because of previous *unpleasant experiences*.

**Table 3:** The attributes of various published observational instruments for identifying and/or assessing behaviour in domestic equids.

	**Authors**	**Year**	**Type of ** **Instrument**	**Capacity for use ** **free from human ** **influences**	**Capacity for use in ** **human–horse ** **interactions**	**Utility for logging ** **conflict** **behaviours**	**Designed for ** **longitudinal ** **study**	**Vulnerability to reporter biases**
Equid play ethogram	McDonnell and Poulin [113]	2002	Ethogram	✓	×	×	✓	✓
Agonistic ethogram of equid bachelor band	McDonnell and Haviland [114]	1995	Ethogram	✓	×	×	✓	✓
Equine Discomfort Ethogram posture and whole-body behaviours	Torcivia and McDonnell [115]	2021	Ethogram	✓	?	✓	×?	✓
Ridden Horse pain Ethogram (RHpE)	Dyson [27]	2018	Ethogram	×	✓	✓	✓	✓
Horse Grimace Scale (HGS)	Dalla Costa, et al. [116]	2014	Scale	✓	✓	×	×	✓
The Horse Chronic Pain Scale (HCPS)	van Loon and Macri [117]	2021	Scale	✓	×	×	×	✓
Musculoskeletal Pain Scale (MPS)	Auer, et al. [118]	2024	Scale	✓	×	×	✓	✓
E-BARQ	Fenner, et al. [119]	2020	Online survey	×	✓	✓	✓	✓

(Key: × for absence of attribute; ✓ for presence of attribute; ? for currently unknown/potential).

## References

[1] Campbell M.L. (2021). An ethical framework for the use of horses in competitive sport: Theory and function. Animals.

[2] Douglas J., Owers R., Campbell M.L. (2022). Social licence to operate: What can equestrian sports learn from other industries?. Animals.

[3] Dyson S., Pollard D. (2022). Application of the Ridden Horse Pain Ethogram to horses competing in British Eventing 90, 100 and Novice one-day events and comparison with performance. Animals.

[4] McLean A.N., McGreevy P.D. (2010). Horse-training techniques that may defy the principles of learning theory and compromise welfare. J. Vet. Behav..

[5] Dyson S., Berger J., Ellis A.D., Mullard J. (2018). Development of an ethogram for a pain scoring system in ridden horses and its application to determine the presence of musculoskeletal pain. J. Vet. Behav..

[6] Brown S.M., Connor M. (2017). Understanding and application of learning theory in UK-based equestrians. Anthrozoös.

[7] Luke K.L., McAdie T., Warren-Smith A.K., Rawluk A., Smith B.P. (2023). Does a working knowledge of learning theory relate to improved horse welfare and rider safety?. Anthrozoös.

[8] Warren-Smith A.K., McGreevy P.D. (2008). Equestrian coaches’ understanding and application of learning theory in horse training. Anthrozoös.

[9] Doherty O., McGreevy P.D., Pearson G. (2017). The importance of learning theory and equitation science to the veterinarian. Appl. Anim. Behav. Sci..

[10] Pearson G., Reardon R., Keen J., Waran N. (2021). Difficult horses–prevalence, approaches to management of and understanding of how they develop by equine veterinarians. Equine Vet. Educ..

[11] Pearson G., Connor M., Keen J., Reardon R., Waran N. (2021). Incorporation of equine learning theory into the undergraduate curriculum. J. Vet. Med. Educ..

[12] Urquiza-Haas E.G., Kotrschal K. (2015). The mind behind anthropomorphic thinking: Attribution of mental states to other species. Anim. Behav..

[13] Bradshaw J., Casey R. (2007). Anthropomorphism and anthropocentrism as influences in the quality of life of companion animals. Anim. Welf..

[14] Minero M., Dalla Costa E., Dai F., Canali E., Barbieri S., Zanella A., Pascuzzo R., Wemelsfelder F. (2018). Using qualitative behaviour assessment (QBA) to explore the emotional state of horses and its association with human-animal relationship. Appl. Anim. Behav. Sci..

[15] Prato-Previde E., Basso Ricci E., Colombo E.S. (2022). The Complexity of the Human–Animal Bond: Empathy, Attachment and Anthropomorphism in Human–Animal Relationships and Animal Hoarding. Animals.

[16] Proctor H. (2012). Animal sentience: Where are we and where are we heading?. Animals.

[17] Mota-Rojas D., Mariti C., Zdeinert A., Riggio G., Mora-Medina P., del Mar Reyes A., Gazzano A., Domínguez-Oliva A., Lezama-García K., José-Pérez N. (2021). Anthropomorphism and its adverse effects on the distress and welfare of companion animals. Animals.

[18] Dyson S., Pollard D. (2023). Application of the Ridden Horse Pain Ethogram to 150 Horses with Musculoskeletal Pain before and after Diagnostic Anaesthesia. Animals.

[19] McGreevy P.D. (2007). The advent of equitation science. Vet. J..

[20] Auty I. (2001). The BHS Manual of Equitation: The Training of Horse and Rider.

[21] Belton C. (2006). German National Equestrian Federation. The Principles of Riding.

[22] Ritter T. (2011). Dressage Principles Based on Biomechanics.

[23] Decarpentry G., Bartle N. (2012). Academic Equitation: A Training System Based on the Methods of D’Aure, Baucher and L’Hotte.

[24] Steinbrecht G., von Heydebreck H., Steinkraus W. (2017). The Gymnasium of the Horse.

[25] Karl P. (2010). The Art of Riding: Classical Dressage to High School—Odin at Saumur.

[26] Hamilton K.L., Lancaster B.E., Hall C. (2022). Equine conflict behaviors in dressage and their relationship to performance evaluation. J. Vet. Behav..

[27] Dyson S. (2022). The Ridden Horse Pain Ethogram. Equine Vet. Educ..

[28] Cook W., Kibler M. (2019). Behavioural assessment of pain in 66 horses, with and without a bit. Equine Vet. Educ..

[29] Górecka-Bruzda A., Kosińska I., Jaworski Z., Jezierski T., Murphy J. (2015). Conflict behavior in elite show jumping and dressage horses. J. Vet. Behav..

[30] Rolls E.T. (2000). Precis of the brain and emotion. Behav. Brain Sci..

[31] Mills D. (1998). Applying learning theory to the management of the horse: The difference between getting it right and getting it wrong. Equine Vet. J..

[32] Farhoody P. (2012). Behavior analysis: The science of training. Vet. Clin. North Am. Exot. Anim. Pract..

[33] FEI Library. https://inside.fei.org/fei/about-fei/fei-library#fei-dressage-handbook---guidelines-for-judging.

[34] FederationEquestreInternationale (2007). FEI Dressage Handbook Guidelines for Judging.

[35] FEI Dressage Judging Manual. https://inside.fei.org/fei/disc/dressage/rules.

[36] FEI Object and General Principles of Para Dressage. https://inside.fei.org/fei/disc/para-dressage/rules.

[37] Dressage Rules. https://inside.fei.org/fei/disc/dressage/rules.

[38] FEI Para Dressage Rules. https://inside.fei.org/fei/disc/para-dressage/rules.

[39] FEI Driving Rules. https://inside.fei.org/fei/disc/driving/rules.

[40] Wolframm I., Reuter P., Zaharia I., Vernooij J. (2024). In the eye of the beholder—Visual search behavior in equestrian dressage judges. Animals.

[41] FEI Measuring Device for Control of Noseband Tightness Gradually Rolled out at 2025 FEI Events. https://eurodressage.com/2024/10/31/fei-measuring-device-control-noseband-tightness-gradually-rolled-out-2025-fei-events.

[42] (2025). FEI Veterinary Regulations. https://inside.fei.org/content/fei-veterinary-rules.

[43] Mellor D.J. (2020). Mouth pain in horses: Physiological foundations, behavioural indices, welfare implications, and a suggested solution. Animals.

[44] Christensen J.W., Uldahl M. (2024). Oral behaviour during riding is associated with oral lesions in dressage horses—A field study. Appl. Anim. Behav. Sci..

[45] Dyson S., Pollard D. (2021). Application of the ridden horse pain ethogram to elite dressage horses competing in World Cup Grand Prix competitions. Animals.

[46] Kienapfel K., Piccolo L., Cockburn M., Gmel A., Rueß D., Bachmann I. (2024). Comparison of head–neck positions and conflict behaviour in ridden elite dressage horses between warm-up and competition. Appl. Anim. Behav. Sci..

[47] What Are the Animal Welfare Issues Associated with Bull Riding?. https://kb.rspca.org.au/knowledge-base/what-are-the-animal-welfare-issues-associated-with-bull-riding/.

[48] Croney C.C., Reynnells R. (2008). The ethics of semantics: Do we clarify or obfuscate reality to influence perceptions of farm animal production?. Poult. Sci..

[49] Wolframm I.A., Douglas J., Pearson G. (2023). Changing hearts and minds in the equestrian world one behaviour at a time. Animals.

[50] Hall C., Kay R. (2024). Living the good life? A systematic review of behavioural signs of affective state in the domestic horse (Equus caballus) and factors relating to quality of life. Part 2: Horse-human interactions. Anim. Welf..

[51] Oxford English Dictionary. https://www.oed.com/.

[52] FEI Eventing Rules. https://inside.fei.org/fei/disc/eventing/rules.

[53] McGreevy P., Christensen J.W., Von Borstel U.K., McLean A. (2018). Equitation Science.

[54] McGreevy P., McLean A. (2010). Equitation Science.

[55] Kortmulder K. (1998). Displacement behaviour solving a silent contradiction. Acta Biotheor..

[56] Wood-Gush D.G.M. (1983). Conflict and Thwarting. Elements of Ethology: A Textbook for Agricultural and Veterinary Students.

[57] LeDoux J.E. (1997). Emotion, memory and the brain. Sci. Am..

[58] Rey C.N., Thrailkill E.A., Goldberg K.L., Bouton M.E. (2020). Relapse of operant behavior after response elimination with an extinction or an omission contingency. J. Exp. Anal. Behav..

[59] Dawkins R., Krebs J.R. (1979). Arms races between and within species. Proc. R. Soc. London. Ser. B. Biol. Sci..

[60] McGreevy P. (2012). Equine Behavior: A Guide for Veterinarians and Equine Scientists.

[61] Fenner K., Wilson B.J., Ermers C., McGreevy P.D. (2024). Reported Agonistic Behaviours in Domestic Horses Cluster According to Context. Animals.

[62] Mills D.S. (2010). The Encyclopedia of Applied Animal Behaviour and Welfare.

[63] Wood-Gush D. (2012). Elements of Ethology: A Textbook for Agricultural and Veterinary Students.

[64] McGreevy P.D., McLean A., Warren-Smith A.K., Waran N., Goodwin D. (2005). Defining the terms and processes associated with equitation. Proceedings of the 1st International Equitation Science Sysposium 2005.

[65] Joint Position Paper on The Animal Welfare Implications of Animal Behavioural Modification, Training Methods, and Ability to Express Species-Specific Behaviours. https://fve.org/cms/wp-content/uploads/FVE-FEEVA-FECAVA-WSAVA-Behaviour-and-training-position-ADOPTED-1.pdf.

[66] Glossary of Behavioral Terms for Veterinary Medicine. https://www.msdvetmanual.com/behavior/behavioral-medicine-introduction/glossary-of-behavioral-terms-for-veterinary-medicine#Conflict_v3296321.

[67] Sneddon L.U., Elwood R.W., Adamo S.A., Leach M.C. (2014). Defining and assessing animal pain. Anim. Behav..

[68] Carbone L. (2020). Do “Prey Species” Hide Their Pain? Implications for Ethical Care and Use of Laboratory Animals. J. Appl. Anim. Ethics Res..

[69] Bolles R.C. (1970). Species-specific defense reactions and avoidance learning. Psychol. Rev..

[70] Levine M.A., Mills D., McDonnell S. (2005). Domestication and early history of the horse. The Domestic Horse. The Evolution, Development and Management of Its Behaviour.

[71] Pearson G., Waran N., Reardon R.J., Keen J., Dwyer C. (2021). A Delphi study to determine expert consensus on the behavioural indicators of stress in horses undergoing veterinary care. Appl. Anim. Behav. Sci..

[72] Gilam G., Gross J.J., Wager T.D., Keefe F.J., Mackey S.C. (2020). What is the relationship between pain and emotion? Bridging constructs and communities. Neuron.

[73] Lundblad J. (2024). Exploring Facial Expressions in Horses—Biological and Methodological Approaches. Doctorate Thesis.

[74] Deuis J.R., Dvorakova L.S., Vetter I. (2017). Methods used to evaluate pain behaviors in rodents. Front. Mol. Neurosci..

[75] Reid J., Nolan A., Scott E. (2018). Measuring pain in dogs and cats using structured behavioural observation. Vet. J..

[76] Mellor D., Cook C., Stafford K. (2000). Quantifying some responses to pain as a stressor. The Biology of Animal Stress: Basic Principles and Implications for Animal Welfare.

[77] Spasojevic S., Bregun-doronjski A. (2011). A simultaneous comparison of four neonatal pain scales in clinical settings. J. Matern.-Fetal Neonatal Med..

[78] Zwakhalen S.M., Hamers J.P., Abu-Saad H.H., Berger M.P. (2006). Pain in elderly people with severe dementia: A systematic review of behavioural pain assessment tools. BMC Geriatr..

[79] Griffin D.R. (2013). Animal Minds: Beyond Cognition to Consciousness.

[80] Cordier L., Diers M. (2018). Learning and unlearning of pain. Biomedicines.

[81] Tong L., Stewart M., Johnson I., Appleyard R., Wilson B., James O., Johnson C., McGreevy P. (2020). A comparative neuro-histological assessment of gluteal skin thickness and cutaneous nociceptor distribution in horses and humans. Animals.

[82] Dyson S., Bondi A., Routh J., Pollard D., Preston T., McConnell C., Kydd J. (2022). Do owners recognise abnormal equine behaviour when tacking-up and mounting? A comparison between responses to a questionnaire and real-time observations. Equine Vet. Educ..

[83] Casey R. (2007). Clinical problems associated with the intensive management of performance horses. The Welfare of Horses.

[84] Dalla Costa E., Pascuzzo R., Leach M.C., Dai F., Lebelt D., Vantini S., Minero M. (2018). Can grimace scales estimate the pain status in horses and mice? A statistical approach to identify a classifier. PLoS ONE.

[85] McBride S.D., Mills D.S. (2012). Psychological factors affecting equine performance. BMC Vet. Res..

[86] Dyson S., Thomson K. (2022). The recognition of pain and learned behaviour in horses which buck. Equine Vet. Educ..

[87] Zetterberg E., Persson-Sjodin E., Lundblad J., Hernlund E., Rhodin M. (2024). Prevalence of movement asymmetries in high-performing riding horses perceived as free from lameness and riders’ perception of horse sidedness. PLoS ONE.

[88] Takeuchi T., Sugita S. (2007). Histological atlas and morphological features by Nissl staining in the amygdaloid complex of the horse, cow and pig. J. Equine Sci..

[89] Abdallah C.G., Geha P. (2017). Chronic pain and chronic stress: Two sides of the same coin?. Chronic Stress.

[90] Edwards P.T., Smith B.P., McArthur M.L., Hazel S.J. (2019). Fearful Fido: Investigating dog experience in the veterinary context in an effort to reduce distress. Appl. Anim. Behav. Sci..

[91] Lush J., Ijichi C. (2018). A preliminary investigation into personality and pain in dogs. J. Vet. Behav..

[92] Ijichi C., Collins L.M., Elwood R.W. (2014). Pain expression is linked to personality in horses. Appl. Anim. Behav. Sci..

[93] Kelly K.J., McDuffee L.A., Mears K. (2021). The effect of human–horse interactions on equine behaviour, physiology, and welfare: A scoping review. Animals.

[94] Janicka W., Wilk I., Próchniak T. (2023). Does social motivation mitigate fear caused by a sudden sound in horses?. Anim. Cogn..

[95] Lundberg P., Hartmann E., Roth L.S. (2020). Does training style affect the human-horse relationship? Asking the horse in a separation–reunion experiment with the owner and a stranger. Appl. Anim. Behav. Sci..

[96] Hartmann E., Rehn T., Christensen J.W., Nielsen P.P., McGreevy P. (2021). From the horse’s perspective: Investigating attachment behaviour and the effect of training method on fear reactions and ease of handling—A pilot study. Animals.

[97] Hama H., Yogo M., Matsuyama Y. (1996). Effects of stroking horses on both humans’ and horses’ heart rate responses 1. Jpn. Psychol. Res..

[98] Normando S., Haverbeke A., Meers L., Ödberg F., Ibáñez Talegón M., Bono G. (2003). Effect of manual imitation of grooming on riding horses’ heart rate in different environmental situations. Vet. Res. Commun..

[99] Rault J.-L., Waiblinger S., Boivin X., Hemsworth P. (2020). The power of a positive human–animal relationship for animal welfare. Front. Vet. Sci..

[100] Fortin M., Valenchon M., Lévy F., Calandreau L., Arnould C., Lansade L. (2018). Emotional state and personality influence cognitive flexibility in horses (*Equus caballus*). J. Comp. Psychol..

[101] Olczak K., Klocek C., Christensen J.W. (2021). Hucul horses’ learning abilities in different learning tests and ue the association with behaviour, food motivation and fearfulness. Appl. Anim. Behav. Sci..

[102] Harewood E., McGowan C. (2005). Behavioral and physiological responses to stabling in naive horses. J. Equine Vet. Sci..

[103] Lerch N., Cirulli F., Rochais C., Lesimple C., Guilbaud E., Contalbrigo L., Borgi M., Grandgeorge M., Hausberger M. (2021). Interest in humans: Comparisons between riding school lesson equids and assisted-intervention equids. Animals.

[104] Murray R.C., Walters J., Snart H., Dyson S., Parkin T. (2010). How do features of dressage arenas influence training surface properties which are potentially associated with lameness?. Vet. J..

[105] Mejdell C.M., Bøe K.E. (2005). Responses to climatic variables of horses housed outdoors under Nordic winter conditions. Can. J. Anim. Sci..

[106] McGreevy P., Oddie C., Burton F., McLean A. (2009). The horse–human dyad: Can we align horse training and handling activities with the equid social ethogram?. Vet. J..

[107] Gordan M., Krishanan I.A. (2014). A review of BF Skinner’s ‘Reinforcement Theory of Motivation’. Int. J. Res. Educ. Methodol..

[108] McLean A.N., Christensen J.W. (2017). The application of learning theory in horse training. Appl. Anim. Behav. Sci..

[109] Bartlett E., Blackwell E.J., Cameron L.J., Hockenhull J. (2024). Are We on the Same Page? A Review of Horse Training Approaches, Terminology Use, and Method Reporting within the Scientific Literature. Int. J. Equine Sci..

[110] McLean A., Henshall C., Starling M., McGreevy P. (2013). Arousal, Attachment and Affective State. Proceedings of the 9th International Equitation Science Conference.

[111] Mendl M., Paul E.S. (2020). Animal affect and decision-making. Neurosci. Biobehav. Rev..

[112] Perone M. (2003). Negative effects of positive reinforcement. Behav. Anal..

[113] McDonnell S.M., Poulin A. (2002). Equid play ethogram. Appl. Anim. Behav. Sci..

[114] McDonnell S., Haviland J. (1995). Agonistic ethogram of the equid bachelor band. Appl. Anim. Behav. Sci..

[115] Torcivia C., McDonnell S. (2021). Equine discomfort ethogram. Animals.

[116] Dalla Costa E., Minero M., Lebelt D., Stucke D., Canali E., Leach M.C. (2014). Development of the Horse Grimace Scale (HGS) as a pain assessment tool in horses undergoing routine castration. PLoS ONE.

[117] van Loon J.P., Macri L. (2021). Objective assessment of chronic pain in horses using the Horse Chronic Pain Scale (HCPS): A scale-construction study. Animals.

[118] Auer U., Kelemen Z., Vogl C., von Ritgen S., Haddad R., Torres Borda L., Gabmaier C., Breteler J., Jenner F. (2024). Development, refinement, and validation of an equine musculoskeletal pain scale. Front. Pain Res..

[119] Fenner K., Matlock S., Williams J., Wilson B., McLean A., Serpell J., McGreevy P. (2020). Validation of the Equine Behaviour Assessment and Research Questionnaire (E-BARQ): A new survey instrument for exploring and monitoring the domestic equine triad. Animals.

[120] Tinbergen N. (1963). On the aims and methods of ethology. Z. Tierpsychol..

[121] Russell J.A. (2003). Core affect and the psychological construction of emotion. Psychol. Rev..

[122] Canine Arthritis Management Tools. https://caninearthritis.co.uk/how-cam-can-help/tools/.

[123] Hall C., Randle H., Pearson G., Preshaw L., Waran N. (2018). Assessing equine emotional state. Appl. Anim. Behav. Sci..

[124] Brereton J.E., Fernandez E.J. (2022). Investigating unused tools for the animal behavioral diversity toolkit. Animals.

[125] McGreevy P.D., Sundin M., Karlsteen M., Berglin L., Ternström J., Hawson L., Richardsson H., McLean A.N. (2014). Problems at the human–horse interface and prospects for smart textile solutions. J. Vet. Behav..

[126] Bosch S., Serra Bragança F., Marin-Perianu M., Marin-Perianu R., Van der Zwaag B.J., Voskamp J., Back W., Van Weeren R., Havinga P. (2018). EquiMoves: A wireless networked inertial measurement system for objective examination of horse gait. Sensors.

[127] Hardeman A., Serra Bragança F., Swagemakers J.-H., Van Weeren P., Roepstorff L. (2019). Variation in gait parameters used for objective lameness assessment in sound horses at the trot on the straight line and the lunge. Equine Vet. J..

[128] Frippiat T., van Beckhoven C., Moyse E., Art T. (2021). Accuracy of a heart rate monitor for calculating heart rate variability parameters in exercising horses. J. Equine Vet. Sci..

[129] Eckardt F., Münz A., Witte K. (2014). Application of a full body inertial measurement system in dressage riding. J. Equine Vet. Sci..

[130] Dumbell L., Lemon C., Williams J. (2019). A systematic literature review to evaluate the tools and methods used to measure rein tension. J. Vet. Behav..

[131] Kelemen Z., Grimm H., Vogl C., Long M., Cavalleri J.M., Auer U., Jenner F. (2021). Equine activity time budgets: The effect of housing and management conditions on geriatric horses and horses with chronic orthopaedic disease. Animals.

[132] Andersen P.H., Gleerup K., Wathan J., Coles B., Kjellström H., Broomé S., Lee Y., Rashid M., Sonder C., Resenberg E. (2018). Can a Machine Learn to see Horse pain? An Interdisciplinary Approach Towards Automated Decoding of Facial Expressions of Pain in the Horse. Proc. Meas. Behav..

[133] Feighelstein M., Riccie-Bonot C., Hasan H., Weinberg H., Rettig T., Segal M., Distelfeld T., Shimshoni I., Mills D.S., Zamansky A. (2024). Automated recognition of emotional states of horses from facial expressions. PLoS ONE.

[134] Lecorps B., Weary D. (2024). Animal affect, welfare and the Bayesian brain. Anim. Welf..

[135] Mellor D.J., Beausoleil N.J., Littlewood K.E., McLean A.N., McGreevy P.D., Jones B., Wilkins C. (2020). The 2020 five domains model: Including human–animal interactions in assessments of animal welfare. Animals.

[136] McGreevy P., Berger J., De Brauwere N., Doherty O., Harrison A., Fiedler J., Jones C., McDonnell S., McLean A., Nakonechny L. (2018). Using the five domains model to assess the adverse impacts of husbandry, veterinary, and equitation interventions on horse welfare. Animals.

[137] König von Borstel U., McGreevy P.D. (2014). Behind the vertical and behind the times. Vet. J..

[138] Can Technology Assist Dressage with Public Trust and with Objective Measurements?. https://eurodressage.com/2024/11/03/can-technology-assist-dressage-public-trust-and-objective-measure.

[139] Boot M., McGreevy P.D. (2013). The X files: Xenophon re-examined through the lens of equitation science. J. Vet. Behav..

[140] Bateson M., Martin P. (2021). Measuring Behaviour: An Introductory Guide.

[141] Pierard M., McGreevy P., Geers R. (2019). Reliability of a descriptive reference ethogram for equitation science. J. Vet. Behav..

